# Should Blind Judokas and Partially Sighted Judokas Compete Together? A Reflection from the Study of the Temporal Structure of Combat

**DOI:** 10.5114/jhk/163281

**Published:** 2023-07-06

**Authors:** Jesús Antonio Gutiérrez-Santiago, Iván Prieto-Lage, Adrián Paramés-González, Juan Carlos Argibay-González, Xoana Reguera-López-de-la-Osa, Alfonso Gutiérrez-Santiago

**Affiliations:** 1 Observational Research Group, Faculty of Education and Sport, Universidade de Vigo, Pontevedra, Spain.; 2 Education, Physical Activity and Health Research Group (Gies10-DE3), Galicia Sur Health Research Institute (IIS Galicia Sur), SERGAS-UVIGO, Spain.

**Keywords:** judo, time-motion, visual impairment, sport class

## Abstract

The investigation of the temporal structure of Para judo combat according to sport classes or visual categories (B1, B2 and B3) has only been carried out in women. The objectives of the study were to analyze the differences in the temporal structure of combat between the male para-judokas sport classes, and to determine whether blind judokas and partially sighted judokas should compete together. All para-judokas who competed in the 2018 IBSA Judo World Championships (172 men) participated in the study. Using observational methodology, we analyzed all the combats (n = 232). To obtain the results, we used different analysis techniques: descriptive, one-way ANOVA, t-test for independent samples and effect size. The level of significance established for the study was ρ ≤ 0.05. The results indicate that during pauses, time dedicated to displacement was increased when there was a B1 judoka in the pairing, causing pauses to be significantly longer. Coaches should take this into account. In conclusion, there are differences between particular visual categories. B1 judokas have longer pauses, perform more ground fighting sequences and win fewer combats. The competition is not on equal terms. More research is needed in order to determine if new regulations have modified the combat temporality.

## Introduction

Research on elite Para sport has been scarce in comparison to healthy elite athletes (Perret, 2017). Not many studies on high performance modalities of Paralympic sports have been developed so far (Griggs et al., 2020). This is also the case of Para judo, a popular discipline with more than 500 para-judokas competing at the highest level (Gutiérrez-Santiago et al., 2020b). Until 2022, para-judokas were classified into three sport classes depending on their visual impairment (namely: B1: blind; B2: severely impaired vision; and B3: moderate to poor vision). The IBSA judo classification rules (IBSA, 2022) have been revised lately. Thus, the sport classes (B1, B2 and B3) have changed from three to two, specifically, J1 for blind and J2 for partially sighted judokas.

A primary goal of the International Paralympic Committee is to enable Para athletes to achieve sporting excellence (Mann et al., 2021). This entails conducting research that may contribute to scientific evidence on the performance determinants of success for these athletes. Some of these factors are linked to Para judo and, therefore, deserve further investigation. For example, different authors have studied performance factors linked to Para judo, such as the number of medals won (Kons et al., 2018, 2019; Krabben et al., 2018), scores (Kons et al., 2018, 2019, 2021b), win ratio (Kons et al., 2021b; Mashkovskiy et al., 2019), penalties (*shido*), efficiency index and technical variation ( Karimizadeh et al., 2020; Kons et al., 2018, 2019), athletes’ ranking score (Kons et al., 2021a), power performance (Loturco et al., 2017) and system of attacks (Calmet et al., 2016). All those investigations conclude that B1 judokas face a disadvantage compared to B2/B3 judokas. Also, another performance factor linked to Para judo is the time-motion, thus it is fundamental to establish a timing structure for para-judokas due to the fact that it has been shown that they need longer pauses as a result of their disability, which means that their recovery period is also longer (Gutiérrez-Santiago et al., 2011). Knowing this time structure would allow coaches to provide individualized training for their athletes (Gutiérrez-Santiago et al., 2022).

Previous studies have shown that para-judokas, compared to sighted judokas, perform shorter fight sequences, longer pause sequences and have more recovery time (Gutiérrez-Santiago et al., 2012). Gender and weight classes were also found to be variables influencing the temporal structure of combat (Gutiérrez-Santiago et al., 2011, 2013). However, since the rules of judo have recently undergone several changes modifying the time structure of the combat (Brabec et al., 2022; Samuel et al., 2020), more up-to-date research is needed. Recent studies have reconfirmed that sex and weight categories remain variables that affect the temporal structure of combat (Gutiérrez-Santiago et al., 2022). Nevertheless, only few investigations have had sport classes as a study variable, and they only included women in their sample, concluding that B1 judoka combats were shorter and that those para-judokas competed under inferior conditions (Gutiérrez-Santiago et al., 2020a). All this leads us to detect an important gap in this field of study, since the investigation of the temporal structure of combat as a function of sport classes has not yet been carried out in men.

Therefore, the objectives of the present study were to analyze the differences in the temporal structure of combat between the sport classes of male para-judokas, and to determine whether blind judokas and partially sighted judokas should compete together.

## Methods

### Design

This study aimed to determine the temporal structure of male combat in blind and partially sighted judokas that participated in the 2018 IBSA Judo World Championship. An observational methodology was used for analysis (Anguera et al., 2018).

The observational design (Anguera et al., 2011) used was nomothetic (in all combats), follow-up (behaviors in the judo match were evaluated throughout the championship), and multidimensional (a concurrence of behaviors was found). Several decisions about the participants, the observational and recording instruments as well as the analytical process are derived from the present design.

### Participants

All the participants were men para-judokas who competed in the IBSA Judo World Championship held in Odivelas-Lisbon (Portugal) in 2018 (n = 172 men in the senior category). Their distribution by sport classes was as follows: B1 = 40, B2 = 62 and B3 = 70. The study followed the ethical principles of the Declaration of Helsinki and the permission for carrying out the investigation was obtained from the IBSA. The study was approved by the Ethics Committee of the Faculty of Education and Sport Science (University of Vigo, Application 01/1019).

### Instruments

The observation instrument used in this study had been used in previous research (Gutiérrez-Santiago et al., 2020a, 2022). Its complete description is provided in Table 1. The validity of the construction of the observational instrument was affected through its coherence with the theoretical framework (based on previous research). Moreover, two experts in observational methodology and judo agreed with the instrument, reaching a level of agreement of 97%.

**Table 1:** Observational Instrument.

Criteria	Category	Code	Description
Visual pairings	B1 vs. B2	B1-B2	B1 judoka competes against a B2 judoka
	B1 vs. B3	B1-B3	B1 judoka competes against a B3 judoka
	B2 vs. B3	B2-B3	B2 judoka competes against a B3 judoka
	B1 vs. B1	B1-B1	B1 judoka competes against a B1 judoka
	B2 vs. B2	B2-B2	B2 judoka competes against a B2 judoka
	B3 vs. B3	B3-B3	B3 judoka competes against a B3 judoka
Moment in the Combat	1^st^ minute	1m	Between 0’’ and 60’’
	2^nd^ minute	2m	Between 61’’ and 120’’
	3^rd^ minute	3m	Between 121’’and 180’’
	4^th^ minute	4m	Between 181’’ and 240’’
	Golden Score	GS	Golden Score – Extra Time
STANDING Fight Sequences	1^st^ standing sequence	STD1	1^st^ sequence of a standing fight
	2^nd^ standing sequence	STD2	2^nd^ sequence of a standing fight
	3^rd^ standing sequence	STD3	3^rd^ sequence of a standing fight
	4^th^ standing sequence	STD4	4^th^ sequence of a standing fight
	Remaining standing sequence	etc.	Until the maximum sequences occur
PAUSE Sequence	1^st^ pause sequence	PA1	1^st^ pause sequence
	2^nd^ pause sequence	PA2	2^nd^ pause sequence
	3^rd^ pause sequence	PA3	3^rd^ pause sequence
	Remaining pause sequence	etc.	Until the maximum sequences occur
Movements during the pause	Displacement	DISPL.	Time spent searching for the judoka to take him/her to the starting position and/or moving to the starting position.
	Grip	GRIP	Time spent from the starting position and gripping until the referee declares *hajime* after a pause.
	Other	OTHER	Time spent on other situations: belt tying, penalties, video checking, etc.
GROUND Fight Sequence	1^st^ ground sequence	GND1	1^st^ ground fight sequence
	2^nd^ ground sequence	GND2	2^nd^ ground fight sequence
	3^rd^ ground sequence	GND3	3^rd^ ground fight sequence
	Remaining ground sequence	Etc.	Until the maximum sequences occur

The data used for analysis were registered using LINCE v.1.4 software (Gabin et al., 2012).

### Procedures

The videos analyzed in the present study were recorded in the location where the competition was held using three Sony model HDR-PJ410 cameras. Following the indications of other investigations (Miarka et al., 2018), each camera was used to record videos in a single combat area, so that ecological validity could be guaranteed.

After attending a training course on the use of previously mentioned instruments, two expert evaluators used the Lince program to observe and record the data of the combats. In order to guarantee the thoroughness of the registration process (Blanco-Villaseñor and Anguera, 2000), the quality of the recorded data was controlled by the concordance calculations of intra and interobservers using the Cohen’s kappa coefficient (Cohen, 1968) calculated with LINCE software. The intra-observer concordance was used in previous combats that did not belong to the definitive sample, in an amount that adds up to one third of the final sample (n = 77), reaching a 0.96 kappa value in observer 1 and 0.94 in observer 2. Subsequently, the inter-observer concordance was calculated, obtaining a 0.91 kappa value.

After a registration of all the combats, an MS-Excel file with the sequentiality of all the codes of the recorded behaviors was obtained, alongside their temporality and duration expressed in frames. The versatility of this file allowed us to carry out successive transformations for the different analyses (Gutiérrez-Santiago et al., 2011).

### Data Analysis

The IBM-Statistical Package for Social Sciences, version 20.0 (IBM-SPSS Inc., Chicago, IL, USA), was used to perform all the data analysis. A stratified descriptive analysis by visual categories was carried out for each of the variables under study using measures of central tendency (mean, standard deviation and 95% confidence intervals). The Kolmogorov-Smirnov test confirmed the sample normality. The present research used one-way ANOVA, applying a post hoc Tukey-b test when statistically significant differences occurred, in order to detect differences between visual categories (B1, B2 and B3), comparing the means of the study variables between all competition parings (B1 vs. B1, B1 vs. B2, B1 vs. B3, B2 vs. B3, B2 vs. B2 and B3 vs. B3). Additionally, taking into consideration the claims of other authors (Krabben et al., 2018, 2019; Mashkovskiy et al., 2019) indicating differences between blind judokas (B1) and partially sighted judokas (B2 and B3), we restructured the data to obtain these groups: judoka B1 combats and combats where there were only B2 or B3 judokas. The comparison of the mean values of these two groups was carried using a *t*-test for independent samples.

In order to determine the visual category that won the most combats, we followed the procedure of other authors (Krabben et al., 2018, 2019; Mashkovskiy et al., 2019), thus, combats between judokas from the same visual category were excluded from the analysis. The difference of victories among visual categories in different parings (B1 vs. B2, B1 vs. B3, B2 vs. B3) was established by the Chi-square.

In all statistical tests, a level of significance of *p* ≤ 0.05 was considered. In addition, we analyzed the effect size through Cohen’s *d* (Cohen, 1988) to determine differences between combats with and without B1 judokas.

## Results

All male competition combats (n = 232) were analyzed. To study differences among visual categories (B1, B2 and B3), a comparison was made between all possible competition parings (B1 vs. B1, n = 16, 6.9%; B1 vs. B2, n = 27, 11.6%; B1 vs. B3, n = 32, 13.8%; B2 vs. B3, n = 80, 34.5%; B2 vs. B2, n = 28, 12.1% and B3 vs. B3, n = 49, 21.1%), finding that statistically significant differences existed in six study variables (Table 2). B1 vs. B2 paring had the most total pause time during the combat, and B3 vs. B3 paring had the least pause time. Displacement time during the total pause time was clearly increased when there was a B1 judoka in paring. B1 vs. B2 and B1 vs. B1 parings clearly spent more time on gripping during the total pause time than the rest of pairings. The longest pause sequences were in B1 vs. B1 pairings and the shortest in B2 vs. B3. During a pause sequence, displacement time was increased when there was a B1 judoka in the pairing, and the gripping time was higher in B1 vs. B2 and B1 vs. B1 pairings.

**Table 2:** Descriptive analysis based on the different combat pairings, ANOVA and the degree of significance of the sequential and temporary variables of judo combats of men with visual impairment.

		Visual pairings							
Study variables	Descriptions	B1B2	B1B3	B2B3	B1B1	B2B2	B3B3	ANOVA	
Total Combat Time	Mean (s)	355.59	203.34	266.96	303.00	247.39	215.10	F	1.932
	SD (s)	287.47	197.54	236.30	250.67	190.12	185.61	g/l	5
	95% CI -Low (s)	241.87	132.12	214.38	169.43	173.67	161.79	Sig	0.090
	95% CI -Up (s)	469.31	274.56	319.55	436.57	321.11	268.42		
Total Standing Time	Mean (s)	111.19	69.81	101.01	87.38	88.36	77.39	F	1.735
	SD (s)	69.45	56.09	82.49	62.96	64.49	61.64	g/l	5
	95% CI -Low (s)	83.71	49.59	82.65	53.82	63.35	59.68	Sig	0.127
	95% CI -Up (s)	138.66	90.03	119.37	120.93	113.36	95.09		
Total Ground Time	Mean (s)	57.95	46.18	43.91	52.46	35.41	37.31	F	1.102
	SD (s)	52.50	36.13	37.58	38.95	35.66	33.80	g/l	5
	95% CI -Low (s)	32.64	30.16	34.67	28.93	19.60	26.35	Sig	0.361
	95% CI -Up (s)	83.25	62.20	53.15	76.00	51.22	48.27		
Total Fight Time	Mean (s)	151.96	101.56	137.24	130.00	116.18	107.08	F	1.617
	SD (s)	103.66	81.10	99.39	91.38	77.85	83.38	g/l	5
	95% CI -Low (s)	110.96	72.32	115.12	81.31	85.99	83.13	Sig	0.157
	95% CI -Up (s)	192.97	130.80	159.35	178.69	146.37	131.03		
Total Pause Time	Mean (s)	250.23	141.83	142.89	213.23	147.16	129.07	F	2.991
	SD (s)	187.17	124.63	144.58	158.07	116.12	106.50	g/l	5
	95% CI -Low (s)	167.24	87.93	109.16	117.71	99.23	95.46	Sig	0.013^a^
	95% CI -Up (s)	333.21	195.72	176.62	308.75	195.09	162.69		
Total Pause Displacement Time	Mean (s)	114.14	68.30	64.42	106.62	58.48	51.02	F	4.401
	SD (s)	88.47	57.85	62.41	83.34	36.73	40.64	g/l	5
	95% CI -Low (s)	74.91	43.29	49.86	56.26	43.32	38.20	Sig	0.001^b^
	95% CI -Up (s)	153.36	93.32	78.99	156.98	73.64	63.85		
Total Pause Gripping Time	Mean (s)	114.45	56.65	55.75	84.00	51.84	56.66	F	4.922
	SD (s)	91.34	49.26	47.43	59.41	36.11	51.17	g/l	5
	95% CI -Low (s)	73.95	35.35	44.69	48.10	36.93	40.51	Sig	0.000^c^
	95% CI -Up (s)	154.95	77.95	66.82	119.90	66.75	72.81		
Total Other Pause Time	Mean (s)	25.32	25.73	34.27	35.00	43.76	29.88	F	0.402
	SD (s)	23.03	28.09	56.36	29.44	72.55	31.43	g/l	5
	95% CI -Low (s)	14.21	10.18	18.08	10.39	10.74	17.19	Sig	0.847
	95% CI -Up (s)	36.42	41.29	50.46	59.61	76.79	42.58		
Total Standing Sequences	Mean	7.85	5.22	6.50	6.06	5.68	5.29	F	1.310
	SD	6.43	4.81	5.29	4.58	3.29	4.19	g/l	5
	95% CI -Low	5.31	3.48	5.32	3.62	4.40	4.08	Sig	0.260
	95% CI -Up	10.39	6.95	7.68	8.50	6.95	6.49		
Total Ground Sequences	Mean	5.05	4.23	3.73	4.31	3.00	3.26	F	1.439
	SD	4.12	3.16	2.74	2.93	2.51	2.79	g/l	5
	95% CI -Low	3.07	2.83	3.05	2.54	1.89	2.35	Sig	0.213
	95% CI -Up	7.04	5.63	4.40	6.08	4.11	4.16		
Total Pause Sequences	Mean	8.64	5.91	6.14	6.46	5.40	5.20	F	1.576
	SD	6.43	4.71	5.35	4.56	3.00	4.23	g/l	5
	95% CI -Low	5.78	3.88	4.89	3.71	4.16	3.86	Sig	0.169
	95% CI -Up	11.49	7.95	7.39	9.22	6.64	6.53		
Standing Sequence Time	Mean (s)	17.23	17.75	16.63	18.11	16.40	14.64	F	0.587
	SD (s)	8.17	13.83	9.54	10.30	8.84	7.52	g/l	5
	95% CI -Low (s)	14.00	12.76	14.50	12.62	12.97	12.48	Sig	0.710
	95% CI -Up (s)	20.46	22.73	18.75	23.59	19.83	16.80		
Ground Sequence Time	Mean (s)	12.32	11.39	12.24	11.65	11.35	12.33	F	0.136
	SD (s)	5.68	5.51	7.03	4.74	4.99	7.74	g/l	5
	95% CI -Low (s)	9.58	8.95	10.51	8.78	9.13	9.82	Sig	0.984
	95% CI -Up (s)	15.06	13.84	13.97	14.51	13.56	14.84		
Pause Sequence Time	Mean (s)	28.88	24.74	22.66	31.31	26.52	25.32	F	3.479
	SD (s)	5.66	7.73	7.54	9.03	11.34	10.17	g/l	5
	95% CI -Low (s)	26.37	21.40	20.91	25.85	21.84	22.11	Sig	0.005^d^
	95% CI -Up (s)	31.39	28.09	24.42	36.76	31.20	28.53		
Pause-Displacement Sequence Time	Mean (s)	13.05	11.50	9.87	15.46	10.75	10.07	F	11.962
	SD (s)	2.59	3.09	2.92	3.04	2.82	2.65	g/l	5
	95% CI -Low (s)	11.90	10.16	9.19	13.62	9.59	9.23	Sig	0.000^b^
	95% CI -Up (s)	14.20	12.84	10.56	17.30	11.92	10.91		
Pause-Grip Sequence Time	Mean (s)	13.25	10.26	9.94	12.93	9.73	11.90	F	2.836
	SD (s)	4.73	4.25	4.19	3.93	4.32	6.43	g/l	5
	95% CI -Low (s)	11.16	8.42	8.96	10.56	7.94	9.87	Sig	0.017
	95% CI -Up (s)	15.35	12.10	10.91	15.31	11.51	13.93		
Pause-Other Sequence Time	Mean (s)	3.00	4.53	4.41	4.39	7.12	5.15	F	1.052
	SD (s)	2.43	4.72	6.16	4.58	9.44	4.65	g/l	5
	95% CI -Low (s)	1.83	1.92	2.64	0.56	2.82	3.27	Sig	0.390
	95% CI -Up (s)	4.17	7.14	6.18	8.22	11.42	7.03		

Notes: a. B1B2 shows significant differences from the rest of the pairings and B3B3 also. b. When there is a B1, more time is spent in the displacement. c. B1B2 shows significant differences from the rest of the pairings as well as B1B2/B1B1. d. B1B1 shows significant differences from the rest of the pairings as well as B2B3.

The sequential and temporal variables of judo combats of two groups are presented in Table 3 as follows: combats involving a B1 judoka (n = 75) and combats involving only B2 or B3 judokas (n = 157). Comparison between the two groups showed significant differences in seven study variables. The effect size analysis (Cohen’s *d*) indicated that differences between the above mentioned groups were between small and large depending on the study variable. Thus, differences between both groups were moderate in the total pause time and in the time of displacement and grip during the total pause time, being clearly greater in B1 judokas. Differences were small in the total ground and pause sequences and in the pause sequence time, values of these variables were higher in B1 judokas. Finally, differences were large in the displacement time during a pause sequence in that they were clearly higher in B1 judokas.

**Table 3:** General descriptive analysis of combats with and without B1, t-test, degree of significance and the effect size of the sequential and temporary parameters of judo combat of men with visual impairment.

Study variables	B1				NO B1							
			95% CI				95% CI		Comparison		Cohen´s *d*	
	Mean	SD	Low	Up	Mean	SD	Low	Up	t	Sig.	*d*	r
Total Combat Time	290.15	253.13	230.24	350.07	243.35	212.62	210.25	276.44	1.361	0.176		
Total Standing Time	91.80	64.51	76.53	107.07	89.83	73.57	78.38	101.28	0.195	0.846		
Total Ground Time	52.29	43.25	40.25	64.33	40.40	35.86	34.16	46.65	1.898	0.059		
Total Fight Time	130.10	93.95	107.86	152.34	122.20	91.27	108.00	136.41	0.602	0.548		
Total Pause Time	206.87	163.56	162.66	251.09	137.77	128.06	116.52	159.01	2.817	0.006*	0.498	0.218
Total Pause Displacement Time	97.96	78.46	76.75	119.17	58.71	52.42	50.02	67.41	3.426	0.001*	0.650	0.278
Total Pause Gripping Time	87.95	74.19	67.89	108.00	54.68	46.22	47.01	62.35	3.100	0.003*	0.600	0.260
Total Other Pause Time	27.83	25.88	19.66	36.00	34.86	54.55	23.86	45.85	−0.787	0.433		
Total Standing Sequences	6.55	5.54	5.24	7.86	5.89	4.64	5.17	6.62	0.933	0.352		
Total Ground Sequences	4.62	3.48	3.65	5.58	3.44	2.70	2.97	3.91	2.184	0.032*	0.400	0.178
Total Pause Sequences	7.33	5.48	5.85	8.81	5.65	4.66	4.88	6.43	2.149	0.033*	0.340	0.151
Standing Sequence Time	17.96	11.19	15.31	20.61	15.86	8.86	14.49	17.24	1.529	0.128		
Ground Sequence Time	11.66	5.26	10.20	13.13	12.16	6.91	10.95	13.36	−0.464	0.643		
Pause Sequence Time	28.03	7.79	25.92	30.14	24.12	9.11	22.61	25.63	2.810	0.005*	0.450	0.196
Pause-Displacement Sequence Time	13.08	3.23	12.20	13.95	10.11	2.82	9.64	10.58	6.344	0.000*	1.010	0.412
Pause-Grip Sequence Time	12.02	4.63	10.77	13.27	10.50	4.98	9.67	11.33	1.958	0.052		
Pause-Other Sequence Time	3.86	3.82	2.65	5.06	5.17	6.66	3.83	6.51	−1.183	0.239		

Notes: * p ≤ 0.05. Expression of the effect size: d y r, d < 0.2 (null), d = 0.2–0.49 (small), d = 0.5–0.80 (moderate) and d > 0.8 (large).

The result of combats is another variable that determines whether there was inequality between different visual categories. For this purpose, we excluded from this analysis combats between judokas of the same visual category. In combats between B1 and B2 (n = 27), B1 won 33.3% (n = 9) and B2 won 66.7% (n = 18) of the combats, without any significant differences (χ^2^ = 3.000, *p* = 0.083). In combats between B1 and B3, B1 only won 28.1% (n = 9) and B3 won 71.9% (n = 23) of the combats, with significant differences (χ^2^ = 6.125, *p* = 0.013). In combats between B2 and B3 (n = 80), B2 won 52.5% (n = 42) and B3 won 47.5% (n = 38) of the combats, without any significant differences (χ^2^ = 0.200, *p* = 0.655).

## Discussion

In the present study, we focused on differences between particular visual categories (B1, B2 and B3), and therefore, a comparison was made between all possible pairings in the men’s competition (B1 vs. B1, B1 vs. B2, B1 vs. B3, B2 vs. B3, B2 vs. B2 and B3 vs. B3). Our findings show that there were significant differences in six study variables. These results differ from the female competition where there were no significant differences between different pairings in any of the study variables (Gutiérrez-Santiago et al., 2020a). Our findings indicate that pauses in men were conditioned by the visual class. Thus, the total pause time, the time of displacement as well as grip during these pauses, pause sequence time and the time of displacement and grip in a pause sequence, in a general way was higher in pairings where B1 judokas were present, especially in B1 vs. B2 and B1 vs. B1. This is probably because B1 judokas, as a consequence of their visual impairment, had to be helped by the referee to return to the starting place of the combat, which made them spend more time in the displacement because they were not able to return on their own. Therefore, participation of a B1 judoka will imply more recovery time for himself as well as for his opponent, which is an aspect to be taken into account by coaches.

Redistribution of the data into two groups, combats where B1 judokas were present (B1 combats) and combats where B1 judokas were not present (NO B1 combats), implied that differences found were even more accentuated. Thus, the total pause time and the time of displacement and grip in these pauses was almost double in B1 combats, for the aforementioned reason. These results are totally opposite to those obtained in female athletes (Gutiérrez-Santiago et al., 2020a). This is due to the fact that in women, the results were conditioned by the total combat time, where B1 combats were significantly shorter than NO B1 combats, because B1 judokas lost the combat earlier (Gutiérrez-Santiago et al., 2020a). On the contrary, in men, no significant differences were found in the total combat time between B1 and NO B1 combats, although being longer in B1 judokas. It should also be noted that in the men’s competition, the total pause sequences, pause sequence time and the displacement time in a pause sequence were significantly higher in B1 combats. Meanwhile in females, the total pause sequences were higher in NO B1 combats, and the duration of a pause sequence did not differ between the two groups (Gutiérrez-Santiago et al., 2020a). In addition, it should be noted that in the men’s competition, the total ground sequences were significantly higher in B1 combats. This may be due to B1 judokas resorting to ground sequences more often not to compensate for visual inequality. An opposite trend was observed in females, where B1 combats had fewer ground sequences (Gutiérrez-Santiago et al., 2020a). This circumstance in women was due to the fact that B1 combats were shorter than NO B1 combats because B1 judokas lost the combat earlier (Gutiérrez-Santiago et al., 2020a). This premature loss has also been observed by other authors (Kons et al., 2019, 2021b; Mashkovskiy et al., 2019).

Delving deeper into this aspect on the victory/defeat of the combat, we can point out that during the period 2007–2016, B1 male judokas won 35.1% of the combats when competing against a B2 judoka (Mashkovskiy et al., 2019). The values are very similar to those of our study, where B1 men won 33.3% of the combats, which contrasts with B1 women, who only won 16.7% of the combats (Gutiérrez-Santiago et al., 2020a). In that period (2007–2016), when B1 male athletes competed against B3 athletes, B1 athletes won 36.5% of the combats (Mashkovskiy et al., 2019). In our research, B1 judokas won 28.1% of the combats. These data are somewhat lower than those obtained by B1 women who won 33.3% of the combats (Gutiérrez-Santiago et al., 2020). In addition, male B2 judokas won 51.6% of the combats when competing with a B3 judoka (Mashkovskiy et al., 2019), which is very similar data to those of our study (52.5%) and to 55.3% of victories achieved by B2 women (Gutiérrez-Santiago et al., 2020a).

All this seems to indicate that the temporality of the combat associated with the pauses is clearly different between B1 judokas and judokas with some visual capability (B2/B3). Thus, B1 judokas spend more time in moving to the starting point of the fight (being assisted by the main referee), while B2/B3 judokas are generally able to return by themselves. In addition, the lower number of victories achieved by B1 judokas, an aspect also noted by other authors (Kons et al., 2021b; Mashkovskiy et al., 2019), shows that the competition does not develop under equal conditions for B1 judokas, a circumstance also demonstrated by other authors from another perspective of analysis (Kons et al., 2019b; Krabben et al., 2018, 2019; Mashkovskiy et al., 2019). Moreover, if we consider that B2/B3 judokas presented higher scores, medals, and efficiency than B1 judokas (Kons et al., 2019; Krabben et al., 2018), it is not surprising that a significant number of authors (Krabben et al., 2019; Krabben et al., 2021a, 2021b) have suggested to equalize the competition conditions in order to minimize the impact of impairment on the outcome of the competition (Tweedy and Vanlandewijck, 2011). And therefore, nor do we consider it to be strange that the International Blind Sports Federation made a recent decision to equalize the conditions of the competition by changing the rules (IBSA, 2022). Since 2022, there are only two sport classes (J1 for blind and J2 for partially sighted judokas), with a separate competition where judokas fight with opponents of the same visual class (J1 vs. J1 and J2 vs. J2). Future research should study how these modifications affect the temporal structure of combat.

## Conclusions

Blind judokas (B1) and partially sighted judokas (B2/B3) should not compete together because there are differences between particular visual categories. B1 judokas take longer pauses than B2/B3 judokas. B1 judokas win fewer combats. They also perform more ground fighting sequences as a strategy to compensate for visual inequality. All this shows that the competition is not on equal terms.

Further research is needed to determine whether recent changes in regulations (separate competition by visual categories) have affected the temporality of combat.
